# Analysis of physical activity in psoriatic arthritis: relationship with clinical and analytical parameters and comorbidity—description of the sedentary patient

**DOI:** 10.3389/fmed.2024.1385842

**Published:** 2024-06-24

**Authors:** Esther Toledano, Carolina Cristina Chacón, Olga Compán, Luis Gómez-Lechón, Cristina Hidalgo, Marta Ibañez, Antonio Márquez, Carlos Montilla

**Affiliations:** ^1^San Carlos University Clinical Hospital, Madrid, Spain; ^2^University Hospital of Salamanca, Salamanca, Spain; ^3^University of Salamanca, Salamanca, Spain; ^4^Regional Hospital Francesc De Borja, Gandia, Spain

**Keywords:** arthritis psoriatic, sedentary lifestyle, comorbidity (source: mesh nlm), obesity, physical activity

## Abstract

**Objective:**

This study aimed to relate physical activity and a sedentary lifestyle to clinical, biological, functional, and comorbid parameters in a cohort of patients with psoriatic arthritis (PsA).

**Methods:**

A cross-sectional study was conducted with 232 PsA patients. Physical activity and sedentary lifestyle were obtained using the *International Physical Activity Questionnaire* (IPAQ) questionnaire. The demographic, clinical, and biological variables measured were age, time since PsA diagnosis, smoking, type of treatment used, clinical form, presence of enthesitis, dactylitis (present or past), fatigue, tumor necrosis factor (TNF)-alpha, and interleukin 6 (IL-6). Activity and functionality were measured using the Disease Activity Index for Psoriatic Arthritis (DAPSA) and Health Assessment Questionnaire (HAQ) in peripheral forms, while the Ankylosing Spondylitis Disease Activity Score (ASDAS-PCR) and Bath Ankylosing Spondylitis Functional Index (BASFI) were measured in axial forms. Disease impact was assessed using the Psoriatic Arthritis Impact of Disease (PsAID) questionnaire. Alongside comorbidities, obesity, anxiety, depression [Hospital Anxiety and Depression Scale (HADS)], and sleep quality [Insomnia Severity Index (ISI)] were assessed.

**Results:**

The mean age was 54.6 (SD: 11.4) years, with 54.3% being male. A total of 25.6% of patients were sedentary. Physical activity and sedentary lifestyle were inversely correlated with fatigue, activity, functionality, and disease impact. Within comorbidities, they correlated with anxiety, depression, and insomnia. In addition, physical activity was inversely correlated with obesity. In linear regression analysis, physical activity was found to be related to body mass index (BMI) with a ß coefficient of −0.1 (*p* < 0.04; 95%CI: −194.1–−4.5), and an R^2^ value of 0.11. In logistic regression analysis, a sedentary lifestyle was found to be related to pain, with an odds ratio (OR) of 1.5 (*p* < 0.001; 95%CI:1.1–1.8) and an R^2^ Nagelkerke value of 0.36.

**Conclusion:**

A quarter of the patients were sedentary. Lack of physical activity correlated with worse parameters of clinical activity, functionality, disease impact, and the presence of comorbidities.

## Introduction

Psoriatic arthritis (PsA) is an inflammatory disease with skin and musculoskeletal manifestations that have a significant impact on the patient in terms of pain, functional disability, and emotional disturbances. Among the comorbidities, obesity is common and is often related to the sedentary lifestyle of the patients. In order to evaluate the benefit of reducing sedentary lifestyles through physical activity, numerous studies have been conducted to assess its effect on disease activity and other symptoms such as fatigue or emotional comorbidities (anxiety, depression, or insomnia) (1–4). The influence of physical activity on disease activity could be explained by the results of previous studies, which have shown that physical activity influences the chronic inflammatory process through the expression of proinflammatory cytokines such as IL-6 and tumor necrosis factor (TNF)-alpha (5–7). In this regard, a meta-analysis involving patients with various chronic inflammatory diseases showed that physical exercise had a slight positive effect on reducing joint inflammation (8). Although more partially, these proinflammatory cytokines may also be involved in the pathogenesis of fatigue and emotional comorbidities in chronic inflammatory diseases (9–11). In this context, high-intensity interval exercise was found to improve fatigue (1). Furthermore, in patients with axial spondyloarthritis, it has been shown to have a potential effect on improving depression (12). Based on the above, the evidence confirms the benefit of physical activity on the different manifestations of PsA. However, few studies have assessed the influence of demographic or clinical factors on reduced physical activity in patients with PsA. In a recent study, it was confirmed that physical activity, as measured by actigraphy, was related to minimal disease activity, age, body mass index, and disease duration. In addition, better mood was related to physical activity (13). Similarly, another study found an inverse relationship between physical activity, measured using accelerometry and a physical activity questionnaire, and disease activity (14).

Against this background, the present study aimed to establish the relationship between physical activity and sedentary lifestyle with demographic factors, clinical activity, fatigue, and comorbidity in a group of patients with PsA.

## Methods

### Type of study

A cross-sectional study was conducted at Salamanca University Hospital (Salamanca, Spain).

### Population

#### Inclusion criteria

We included consecutive patients over 18 years old with a diagnosis of PsA according to the ClASsification criteria for Psoriatic Arthritis (CASPAR) (15) who were seen in outpatient Rheumatology clinics between January 2023 and November 2023 and agreed to participate in the study.

#### Exclusion criteria

1. Patients over 69 years of age, as this is the age limit established by the questionnaire used for the assessment of physical activity.
2. Patients with radiographic damage (erosion or joint space narrowing) to the weight-bearing joints (hip, knee, and foot) or those who had undergone prosthetic replacement surgery (hip or knee) in these joints.
3. Patients with respiratory or cardiac diseases that limit physical activity, according to the clinician’s opinion.

The study was approved by the ethics committee of Salamanca University Hospital (EO 2023 011246—TFG). Patients gave written informed consent before inclusion in the study and for the publication of the results derived from the research.

### Variables assessed

#### Physical activity, demographics, clinical characteristics, and biological parameters

The level of physical exercise was assessed using the *International Physical Activity Questionnaire* (IPAQ) (16, 17). The aforementioned questionnaire assesses physical activity based on three characteristics: intensity (low, moderate, and vigorous), frequency (days per week), and duration (minutes per day). The metabolic equivalents (METs) system is a common procedure to quantify the intensity of physical activity in older adults. A MET is defined as the resting metabolic rate, which is the amount of oxygen consumed at rest while sitting quietly in a chair, approximately 3.5 mL 0_2_/kg/min (1.2 kcal/min for a 70-kg person) (17). METs were the energy expenditure caused by the product of the duration, frequency, and intensity of a given type of exercise. Thus, the MET score corresponding to the type of activity (3.3 for low, 4 for moderate, and 8 for vigorous) was multiplied by the number of days per week the activity is performed and by the minutes spent in the activity per day. The results were published in MET-minutes per week (for example, an individual who indicates they walk 7 days a week for approximately 30 min/day equates to 210 min of activity). According to the IPAQ scoring protocol, MET-minutes/week = 3.3 × walking minutes × walking days in the week, which is equivalent to 693 MET-minutes/week.

We classified patients as sedentary and non-sedentary according to the World Health Organization (WHO) definition (patients with less than 90 min of physical activity per week for sedentary patients and more than 90 min of physical activity per week for non-sedentary patients) (18).

The data were collected on the following variables: age, sex, time since diagnosis, smoking status (smoker/former smoker/never smoker), patients treated with conventional synthetic disease-modifying drugs (scDMARDs), with biological disease-modifying drugs (bDMARDs), or targeted synthetic disease-modifying drugs (tsDMARDs) and patients with failure due to ineffectiveness of bDMARD or tsDMARD at the time of the study. Clinical form of the disease (peripheral, mixed or axial), with axial forms defined as inflammatory low back pain with radiographic damage (sacroiliitis of at least grade 2 according to New York criteria and/or presence of syndesmophytes) (19, 20), the presence of dactylitis (current or past) and the number of involved entheses as assessed by the Modified Maastricht Ankylosing Spondylitis Enthesis Score (mMASES) (21) were also measured. The original MASES (22) focuses on 15 entheseal sites (the bilateral first and seventh costochondral joints, anterior and posterior superior iliac spines, iliac crests, and proximal insertion of the Achilles tendons, as well as the fifth lumbar spinous process), and this has been modified for PsA to include the plantar fascia, with scores ranging from 0 to 15 (21). Fatigue was assessed with a Functional Assessment of Chronic Illness Therapy (FACIT) scale, specifically, the FACIT-fatigue scale, which has been validated for PsA (23) and consists of 13 items assessing self-reported fatigue and its impact on activities of daily living and functioning. Items are rated on a 5-point Likert-type scale from 0 to 4, yielding a total score between 0 and 52, with higher scores indicating less fatigue. Permission was obtained from FACIT.org for the use of the questionnaire in this study.

Due to previously published evidence that there may be a relationship between physical exercise and inflammatory cytokines, we determined serum levels of IL-6 and TNF-alpha (IMMULITE and IMMULITE 1000 systems). This used a solid-phase immunometric assay on Siemens Healthcare Diagnostics Products Ltd. Glyn Rhonwy, Llanberis, Gwynedd, LL55 4EL, United Kingdom.

#### Disease activity, functioning, and disease impact

In patients with peripheral involvement, disease activity was measured using the Disease Activity Index for Psoriatic Arthritis (DAPSA) (24). In the case of patients with axial involvement, we used the Ankylosing Spondylitis Disease Activity Score with CRP (ASDAS-CRP) (25). Functional ability was measured using the Health Assessment Questionnaire-Disability Index (HAQ-DI) for peripheral involvement and the Bath Ankylosing Spondylitis Functional Index (BASFI) for axial involvement, and the impact of the disease was measured using the Psoriatic Arthritis Impact of Disease (PsAID-12) (26, 27).

#### Comorbidities (obesity, anxiety, depression, and insomnia)

Obesity was measured using the body mass index (BMI) (28). BMI is the result of dividing weight measured in kilograms by the square of height measured in meters.

We assessed the emotional factors using the Hospital Anxiety and Depression Scale (HADS) (29). The HADS is a 14-item scale designed to identify people with anxiety and depression among individuals with medical conditions. Scores range from 0 to 21 for each subscale (HADS-D for depression and HADS-A for anxiety) and can be classified into one of the three categories: normal (0–7), borderline abnormal indicating a possible clinical disorder (8–10), and abnormal indicating a probable clinical disorder.

Sleep quality was analyzed using the Insomnia Severity Index (ISI) (30). This is a self-administered questionnaire composed of seven items assessing the nature, severity, and impact of insomnia. Responses are rated on a 5-point Likert-type scale ranging from 0 to 4, referring to the last month. The overall score ranges between 0 and 28 and can be interpreted with cutoff scores as follows: no clinically significant insomnia (0–7), subthreshold insomnia (8–14), moderate severity clinical insomnia (15–21), and severe clinical insomnia (22–28).

### Statistical analysis

Quantitative variables are reported as means and standard deviations, and categorical variables as numbers and percentages. Comparisons between groups were carried out using the Student’s t-test for normally distributed quantitative variables and the Mann–Whitney U-test for ordinal variables or non-normally distributed quantitative variables. Comparisons between more than two groups were performed using a one-factor analysis of variance for normally distributed quantitative variables and the Kruskal–Wallis H-test for ordinal variables or non-normally distributed quantitative variables. Correlations between quantitative variables were assessed with Pearson’s correlation coefficient. *p*-values < 0.05 were considered statistically significant.

Bivariate correlations were calculated between physical activity and DAPSA (and components), HAQ, FACIT-F, PSAID, BMI, HADS-A, HADS-D, and ISI. The linear regression model was performed with exercise as the dependent variable and as independent variables those that had been significant in the univariate analysis and those that, according to the literature, had influenced physical activity (age, sex, BMI, TJC, VAS pain, HADS-A, HADS-D, FACIT-F, and ISI) (13, 14, 31).

On the other hand, two groups of patients were established according to the definition of a sedentary lifestyle, and comparisons were made in demographic variables, clinical characteristics, activity, functionality, impact, and comorbidities between both groups of patients. Similarly, a logistic regression model was performed using sedentary lifestyle as the dependent variable and as independent variables those that had been significant in the univariate analysis and those that, according to the literature, had influenced physical activity (age, sex, enthesitis, TJC, SJC, VAS pain, BMI, HADS-A/HADS-D, FACIT-F, and ISI) (13, 14, 31).

## Results

### Demographic, clinical, and disease-related characteristics of patients with psoriatic arthritis

Two hundred and thirty-two patients participated in the study. Seventy-three patients had low physical activity (32%), 89 had moderate activity (38%), and 70 had high activity (30%). The mean physical exercise was 2232.2 ± 2360.7 METs min-week. A total of 25.9% of the patients were sedentary.

The mean age of the patients was 54.6 (SD: 11.4) years, with a proportion of 54.3% male patients. The duration of the disease was 11.0 (SD: 8.4) years. A total of 67.2% of patients were on methotrexate, whereas 22.8% were on tsDMARDs or bDMARDs. A total of 10.3% of patients who were on treatment with tsDMARDs or bDMARDs had a failure due to inefficacy. The rest of the results are presented in Table 1.

**Table 1:** Demographic, clinical, and disease-related characteristics of patients with psoriatic arthritis.

Total		*n* = 232
		Mean ± standard deviation or number (%)
**Smoking status**		
	Smoke	60 (26)
	Former smoker	108(46)
	Non-smoker	64 (28)
Conventional synthetic DMARDs *N*(%)		185 (80)
	Methotrexate	156 (84)
	Sulfasalazine	14 (8)
	Leflunomide	15(8)
Targeted synthetic DMARDs *N*(%)		8 (3)
Biological DMARDs *N*(%)		48 (21)
	TNF inhibitor	36 (16)
	Other	12 (5)
**Clinical form *N*(%)**		
	Peripheral	211 (91)
	Mixed	21 (9)
	Axial	0 (0)
mMASES		1.4 ± 2.0
Dactylitis *N*(%)		37 (16)
FACIT-Fatigue		36.6 ± 11.0
IL-6 (pg/dL)		3.8 + 3.0
TNF-alpha (pg/dL)		8.6 + 5.7
DAPSA		9.8 ± 6.3
Pain VAS		3.4 ± 2.7
Activity VAS		3.2 ± 2.6
TJC		2.5 ± 1.9
SJC		1.4 ± 0.9
CPR (mg/dL)		0.5 ± 0.7
HAQ		0.5 ± 0.5
ASDAS-CRP^*^		2.1 ± 0.8
BASFI^*^		3.5 ± 2.2
PsAID		3.1 ± 2.0
BMI (kg/m^2^)		26.6 ± 4.2
HADS anxiety		6.9 ± 4.2
HADS depression		4.8 ± 3.7
ISI		8.9 ± 6.3

ASDAS-CRP, Ankylosing Spondylitis Disease Activity Score with C-reactive protein; BASFI, Bath Ankylosing Spondylitis Functional Index; BMI, body mass index; DAPSA, Disease Activity Index for Psoriatic Arthritis; DMARDs, disease-modifying antirheumatic drugs; FACIT, Functional Assessment of Chronic Illness Therapy; HADS, Hospital Anxiety and Depression Scale; HAQ-DI, Health Assessment Questionnaire-Disability Index; IL-6, interlekin-6; ISI, Insomnia Severity Index; mMASES, modified Maastricht Ankylosing Spondylitis Enthesitis; PsAID, Psoriatic Arthritis Impact of Disease; SJC, swollen joint count; TJC, tender joint count; TNF, tumor necrosis factor; VAS, visual analog scale. ^*^In mixed forms (*n* = 21).

### Relationship between physical activity and demographic, clinical, and analytical variables

There was no statistically significant difference in physical activity between sexes (female/male): 2016.7 ± 2388.6 vs. 2414.4 ± 2331.0 METs min-week; *p* = 0.07, smoking habit (smoker/ex-smoker/never smoker): 1850.0 ± 2150.5 vs. 2436.8 ± 2406.8 vs. 2249.1 ± 2462.0 METs min-week; *p* = 0.3, tsDMARDs or bDMARDs (yes/no): 2051. 0 ± 2083.1 vs. 2287.4 ± 2441.9 METs min-week; *p* = 0.7, failure of tsDMARDs or bDMARDs (yes/no): 1918.0 ± 1861.5 vs. 2269.4 ± 2414.4 METs min-week; p = 0. 7, clinical form (peripheral/mixed): 2169.7 ± 2309.2 vs. 2737.5 ± 2742.2 METs min-week; p = 0.3 or presence of dactylitis (yes/no): 2329.4 ± 2229.5 vs. 2213.3 ± 2390.6 METs min-week; *p* = 0.5.

Of the remaining baseline parameters, we only found a correlation between physical activity and fatigue (r: −0.1; *p* < 0.02), but no correlation with age (r: 0.0; *p* = 0.2), time of disease progression (r: 0.0; *p* = 0.7), or the number of affected entheses (r: −0.0; *p* = 0.3). Finally, patients with higher physical activity had higher serum IL-6 concentrations (r: 0.2; *p* < 0.001). However, there was no correlation with serum TNF-alpha concentrations (r: −0.0; *p* = 0.3).

### Relationship between physical activity and disease activity, functionality, and impact

In the peripheral forms, there was a relationship between physical activity and disease activity (DAPSA: r: −0.1; *p* = 0.01). Separated by DAPSA components, physical activity correlated with CRP (r: −0.1; *p* < 0.02), TJC (r: −0.1; *p* < 0.03), and pain VAS (r: −0.1; *p* < 0.02) but not with activity VAS (r: −0.1; *p* = 0.05) nor with SJC (r: 0.1; *p* = 0.8).

In the forms with axial involvement, there were no statistically significant differences between physical activity and axial activity (ASDAS-PCR: r: −0.0; *p* = 0.5).

Regarding functionality, we found a relationship between physical activity and HAQ (r: −0.1; *p* < 0.04) but not with BASFI (r: −0.0; *p* = 0.6). Finally, patients with higher disease impact had lower physical activity (r: −0.1; *p* < 0.03).

### Relationship between physical activity and comorbidities

There was a correlation of physical activity with BMI (r: −0.1; *p* < 0.04), anxiety (HADS-A: r: −0.1; *p* < 0.03), depression (HADS-D: r: −0.1; *p* < 0.03), and sleep quality (ISI: r: −0.2; *p* < 0.01).

In the linear regression model, BMI had a ß coefficient of −0.1 (*p* < 0.04; 95%CI, −194.1–−4.5) with an R^2^ value of =0.11. In the rest of the variables: Age (*p* = 0.1), sex (*p* = 0.1), FACIT-F (*p* = 0.8), TJC (*p* = 0.5), VAS pain (*p* = 0.5), HADS-A (*p* = 0.8), HADS-D (*p* = 0.9), and ISI (*p* = 0.4).

### Relationship between sedentary patients and demographic variables, clinical variables, analytical determinations, disease activity, functionality, impact, and comorbidities

Women were more sedentary compared to men (32.0 vs. 20.6; *p* < 0.04). Sedentary patients had higher levels of fatigue (31.0 ± 11.9 vs. 38.5 ± 10.0; *p* < 0.01), more enthesitis (1.6 ± 1.5 vs. 1.0± 1.5; *p* < 0.001), higher disease activity (12.7 ± 6.5 vs. 8.8 ± 5.9; *p* < 0.001), lower functionality in both peripheral (0.9 ± 0.5 vs. 0.5 ± 0.5; *p* < 0. 001) and axial (3.6 ± 2.6 vs. 2.4 ± 2.3; *p* < 0.01), greater impact of the disease (4.1 ± 2.0 vs. 2.8 ± 1.9; *p* < 0.001), greater HASD-A (8.7 ± 4. 5 vs. 6.3 ± 3.9; *p* < 0.001), greater HADS-D (7.0 ± 4.0 vs. 4.1 ± 3.7; *p* < 0.001), and worse sleep quality (11.9 ± 7.5 vs. 7.7 ± 5.0, *p* < 0.001).

The rest of the comparisons are presented in Table 2.

**Table 2:** Relationship between lifestyles (sedentary or non-sedentary) and demographic variables, clinical variables, analytical determinations, disease activity, functionality, impact, and comorbidities.

Variables deviation or number (%)	Sedentary	No sedentary	*P*
	(*n* = 60)	(*n* = 172)	
Age^*^	54.4 ± 11.3	54.6 ± 11.5	0.9
Time of disease evolution^*^	9.9 ± 7.2	11.4 ± 8.7	0.3
**Smoking status (*N*/%)**			
Smoker	20 (33)	40 (23)	
Former smoker	23 (38)	85 (50)	
Non-smoker	17 (19)	47 (27)	0.2
Targeted synthetic DMARDs or biological DMARDs *N*(%)	16 (26.7)	37(21.5)	0.4
Patients that failed at least one Targeted synthetic DMARDs or biological DMARDs due to inefficacy *N*(%)	8(13.3)	16(9.3)	0.3
Clinical form *N*(%)			0.09
Peripheral	59 (95)	152 (87)	
Mixed	3 (5)	18 (13)	
Dactilitis *N* (%)	10 (16.7)	27 (15.7)	0.8
IL-6(pg/dL)	3.5 ± 2.7	3.9 ± 3.2	0.4
TNF-alpha(pg/dL)	8.6 ± 6.3	8.3 ± 3.2	0.4
PCR (mg/dL)	0.5 ± 0.8	0.4 ± 0.5	0.3
Pain VAS	5.0 ± 2.8	3.1 ± 2.6	0.001
Activity VAS	4.1 ± 2.8	2.9 ± 2.4	0.003
SJC	1.6 ± 0.9	1.3 ± 0.9	0.06
TJC	2.9 ± 1.9	2.4 ± 1.9	0.05
ASDAS-PCR^*^	1.9 ± 0.8	1.7 ± 0.8	0.2
BMI (kg/m^2^)	27.5 ± 5.4	26.3 ± 3.7	0.12

ASDAS-CRP, Ankylosing Spondylitis Disease Activity Score with C-reactive protein; BMI, body mass index; DAPSA, Disease Activity Index for Psoriatic Arthritis; DMARDs, disease-modifying antirheumatic drugs; SJC, swollen joint count; TJC, tender joint count; TNF, tumor necrosis factor; VAS, visual analog scale. ^*^In mixed forms (*n* = 21).

In the logistic regression model, VAS pain: OR: 1.5; *p* < 0.001 (95%CI: 1.1–1.8). R^2^ Nagelkerke:0.36. In the rest of the variables Age (*p* = 0.3), sex (*p* = 0.8), enthesitis (*p* = 0.06), TJC (*p* = 0.1), SJC (*p* = 0.07), BMI (*p* = 0.1), HADS-A/HADS-D (*p* = 0.1), FACIT-F (*p* = 0.9), and ISI (*p* = 0.1).

## Discussion

In our study, physical activity was related to obesity. On the other hand, a sedentary lifestyle was associated with pain intensity. Although there is a multitude of studies evaluating the benefits of increased physical activity (performed through different types of exercise, activity parameters, and comorbidity of joint diseases), there is nevertheless scarce literature on the relationship of disease characteristics to baseline physical activity in patients with PsA. This may be because, due to the cross-sectional methodology of these studies and the bilateral nature of the variables studied, they can be interpreted as both cause and consequence, making it difficult to make conclusive assertions. In the study by McGagh et al., with similar findings to ours, they also found an inverse relationship between physical activity, measured by actigraphy, and BMI. Undoubtedly, a clear bidirectional relationship was established between physical activity and BMI: on the one hand, decreased physical activity is one of the main causes of obesity; on the other hand, obesity in rheumatic diseases limits physical activity because of the pain associated with the mechanical factor, as well as promoting inflammation (31).

Furthermore, we found that, although in most clinical variables related to the disease (number of affected entheses, NAD, NAT, pain, or fatigue) or comorbidities (obesity, anxiety, depression, or insomnia), only pain was related to sedentary lifestyle in the regression analysis. To our knowledge, there is no study of the influence of these characteristics on sedentary behavior in patients with PsA. Similar studies have found similar results to ours: McGagh et al. confirmed that patients who did not reach the therapeutic goal of minimal disease activity (MAE) were less physically active. The authors did not show the relationship between the different items that make up MAE (13). In a recent study by Queiro et al. using data from the Spanish registry of new-onset PsA, low physical activity was predictive of moderate or high disease activity at 2 years (32). Along the same lines, Hernández-Hernández et al., in a cohort of 52 patients measuring physical activity by accelerometry and additionally with the IPAQ, found an inverse relationship between physical activity and higher DAPSA score (14). Although in both our study and that of Hernández-Hernández, this relationship was justified more by pain than inflammation, it has been documented that exercise influences proinflammatory cytokines. Moderate exercise reduces TNF-alpha secretion and intense exercise increases IL-6 levels, which under certain circumstances can decrease TNF-alpha levels (5–7). In our study, although there was an inverse correlation between physical activity and TNF-alpha levels, this was not significant; in contrast, physical activity correlated with IL-6 levels. We cannot make comparisons with other studies because, to our knowledge, the impact of physical activity on these cytokines in patients with PsA has not been studied at present. In forms with axial manifestations, we found no correlation between physical activity or sedentary lifestyle and disease activity.

Hernández-Hernández’s study also found an inverse relationship with functionality. In our study, we found an inverse relationship between physical activity and fatigue. This association has been demonstrated in intervention studies which have shown that high-intensity interval physical exercise can temporarily improve fatigue (1).

Physical activity and a sedentary lifestyle also showed an inverse relationship with disease impact. These results were similar to those obtained in the study by Queiro et al., where physical activity was a predictor of disease impact at 2 years (33).

Among the clinical variables, we found that a sedentary lifestyle was associated with female sex. In previous studies, there have been no similar results so far (13, 14).

Regarding comorbidities, in our study, we found an inverse relationship between physical activity and sleep quality. McGagh et al. suggested that the sleep–sleep relationship may correlate with physical activity (13). In patients with rheumatoid arthritis, this relationship has also been found (34). Regarding anxiety or depression, McGagh et al. related physical activity to mood (13).

Our study has some limitations. As mentioned above, the cross-sectional nature of the study limits our ability to establish causal relationships. On the other hand, the use of questionnaires to establish patients’ physical activity may not be the best method to measure it. However, IPAQ has shown reasonable agreement with other methods such as accelerometry in patients with PsA, rheumatoid arthritis, or healthy adults (14, 34, 35). Although we collected TJC and SJC, it was not specified whether the affected joint could be directly involved with physical activity. Finally, in the regression models, although we included different demographic, clinical, activity-related, and comorbidity-related variables, they did not account for more than 36% of physical activity or sedentary behavior. This may be due to the fact that other variables of a personal nature (love of exercise or lack of time due to work, family responsibilities, etc.) may have a greater influence on physical exercise than those analyzed in this study. It would be interesting to add a small survey reflecting the motivations why the patient does not do more physical activity.

The strengths lie in the inclusion of interleukins (TNF-alpha and IL-6) that have been related to physical activity in other scenarios. Furthermore, we performed the analysis not only in peripheral forms but also incorporated the corresponding measures of activity and functionality in patients with axial manifestations. The relationship between physical activity and so many comorbidities (anxiety, depression, and sleep quality) has also not been studied in similar studies. Finally, to our knowledge, no such study had been carried out in sedentary patients with PsA.

## Conclusion

Physical activity was related to pain, inflammation, and both metabolic and emotional comorbidities. More than 25% of PsA patients are sedentary, so based on the results of our study, we may think that improvement of pain could be achieved by increasing physical activity. However, we should look deeper into the reasons for this lack of physical activity and adopt personalized measures to combat it due to its effects on cardiovascular diseases.

## Data availability statement

The raw data supporting the conclusions of this article will be made available by the authors, without undue reservation.

## Ethics statement

The studies involving humans were approved by the Ethics Committee of Salamanca University Hospital (EO 2023 011248—TFG). The studies were conducted in accordance with the local legislation and institutional requirements. The participants provided their written informed consent to participate in this study. Written informed consent was obtained from the individual(s) for the publication of any potentially identifiable images or data included in this article.

## Author contributions

ET: Conceptualization, Funding acquisition, Investigation, Methodology, Project administration, Resources, Software, Supervision, Validation, Visualization, Writing – review & editing. CC: Data curation, Investigation, Methodology, Writing – review & editing. OC: Data curation, Writing – review & editing. LG-L: Writing – original draft, Writing – review & editing. CH: Conceptualization, Data curation, Formal analysis, Writing – review & editing. MI: Conceptualization, Data curation, Formal analysis, Methodology, Writing – review & editing. AM: Data curation, Methodology, Writing – review & editing. CM: Conceptualization, Data curation, Formal analysis, Funding acquisition, Investigation, Methodology, Project administration, Resources, Software, Supervision, Validation, Visualization, Writing – original draft, Writing – review & editing.
