# Supplementary material for: Analysis of physical activity in psoriatic arthritis: relationship with clinical and analytical parameters and comorbidity—description of the sedentary patient
Source: Front Med (Lausanne). 2024 Jun 24;11:1385842. doi: 10.3389/fmed.2024.1385842 (PMC11229948; doi:10.3389/fmed.2024.1385842)
Supplement: Supplementary file 1 [file Data_Sheet_1.PDF]

FACIT Fatigue Scale (Version 4)

Below is a list of statements that other people with your illness have said are important. **Please circle or mark one number per line to indicate your response as it applies to the past 7 days.**

		Not at all	A little bit	Some-what	Quite a bit	Very much
HI7	I feel fatigued	0	1	2	3	4
HI12	I feel weak all over	0	1	2	3	4
An1	I feel listless ("washed out")	0	1	2	3	4
An2	I feel tired	0	1	2	3	4
An3	I have trouble starting things because I am tired	0	1	2	3	4
An4	I have trouble finishing things because I am tired	0	1	2	3	4
An5	I have energy	0	1	2	3	4
An7	I am able to do my usual activities	0	1	2	3	4
An8	I need to sleep during the day	0	1	2	3	4
An12	I am too tired to eat	0	1	2	3	4
An14	I need help doing my usual activities	0	1	2	3	4
An15	I am frustrated by being too tired to do the things I want to do	0	1	2	3	4
An16	I have to limit my social activity because I am tired	0	1	2	3	4

Copyright 1987, 1997
