# Supplementary material for: Analysis of physical activity in psoriatic arthritis: relationship with clinical and analytical parameters and comorbidity—description of the sedentary patient
Source: Front Med (Lausanne). 2024 Jun 24;11:1385842. doi: 10.3389/fmed.2024.1385842 (PMC11229948; doi:10.3389/fmed.2024.1385842)
Supplement: Supplementary file 2 [file Data_Sheet_2.PDF]

The EULAR Psoriatic Arthritis Impact of Disease: PsAID12 for clinical practice

We want you to indicate how much your psoriatic arthritis impacts your health.
Please tell us how you have been feeling this last week.

1. Pain

Circle the number that best describes the pain you felt due to your psoriatic arthritis during the last week:

None	0	1	2	3	4	5	6	7	8	9	10	Extreme

For office use only

Result x3 ☐

2. Fatigue

Circle the number that best describes the overall level of fatigue due to your psoriatic arthritis you have experienced during the last week:

No fatigue	0	1	2	3	4	5	6	7	8	9	10	Totally exhausted

Result x2 ☐

3. Skin problems

Circle the number that best describes the skin problems including itching you felt due to your psoriatic arthritis during the last week:

None	0	1	2	3	4	5	6	7	8	9	10	Extreme

Result x2 ☐

4. Work and/or leisure activities

Circle the number that best describes the difficulties you had to participate fully in work and/or leisure activities due to your psoriatic arthritis during the last week:

None	0	1	2	3	4	5	6	7	8	9	10	Extreme

Result x2 ☐

5. Functional capacity

Circle the number that best describes the difficulty you had in doing daily physical activities due to your psoriatic arthritis during the last week:

No difficulty	0	1	2	3	4	5	6	7	8	9	10	Extreme difficulty

Result x2 ☐

6. Discomfort

Circle the number that best describes the feeling of discomfort and annoyance with everyday tasks due to your psoriatic arthritis during the last week:

None	0	1	2	3	4	5	6	7	8	9	10	Extreme

Result x2 ☐

7. Sleep disturbance

Circle the number that best describes the sleep difficulties (i.e., resting at night) you felt due to your psoriatic arthritis during the last week:

No difficulty	0	1	2	3	4	5	6	7	8	9	10	Extreme difficulty

Result x2 ☐

8. Coping

Considering your psoriatic arthritis overall, how well did you cope (manage, deal, make do) with your psoriatic arthritis during the last week?

For office use only

Very well	0	1	2	3	4	5	6	7	8	9	10	Very poorly

Result x1 ☐

9. Anxiety, fear and uncertainty

Circle the number that best describes the level of anxiety, fear and uncertainty (for example about the future, treatments, fear of loneliness) due to your psoriatic arthritis you have experienced during the last week:

None	0	1	2	3	4	5	6	7	8	9	10	Extreme

Result x1 ☐

10. Embarrassment and/or shame

Considering your psoriatic arthritis overall, circle the number that best describes the level of embarrassment and/or shame due to your appearance experienced during the last week:

None	0	1	2	3	4	5	6	7	8	9	10	Extreme

Result x1 ☐

11. Social participation

Circle the number that best describes the difficulties you had to participate fully in social activities (including relationships with family and/or people very close to you) due to your psoriatic arthritis during the last week:

None	0	1	2	3	4	5	6	7	8	9	10	Extreme

Result x1 ☐

12. Depression

Circle the number that best describes the level of depression due to your psoriatic arthritis you have experienced during the last week:

None	0	1	2	3	4	5	6	7	8	9	10	Extreme

Result x1 ☐

THANK YOU FOR ANSWERING THIS QUESTIONNAIRE

Final PsAID out of 20

Add up the ☐ and divide by 10:

☐

PsAID12 SCORING AND CALCULATION RULES

The PsAID is calculated based on 12 Numerical rating scales (NRS) questions. Each NRS is assessed as a number between 0 and 10.

Calculation

PsAID final value =
(PsAID1 (pain) NRS value (range 0-10) x 3)
+ (PsAID2 (fatigue) NRS value (range 0-10) x 2)
+ (PsAID3 (skin) NRS value (range 0-10) x 2)
+ (PsAID4 (Work and/or leisure activities) NRS value (range 0-10) x 2)
+ (PsAID5 (function) NRS value (range 0-10) x 2)
+ (PsAID6 (discomfort) NRS value (range 0-10) x 2)
+ (PsAID7 (sleep) NRS value (range 0-10) x 2)
+ (PsAID8 (coping) NRS value (range 0-10) x 1)
+ (PsAID9 (anxiety) NRS value (range 0-10) x 1)
+ (PsAID10 (embarrassment) NRS value (range 0-10) x 1)
+ (PsAID11 (social life) NRS value (range 0-10) x 1)
+ (PsAID12 (depression) NRS value (range 0-10) x 1)

The total is divided by 20.

Thus, the range of the final PsAID value is 0-10 where higher figures indicate worse status.

Missing data imputation

If one of the 12 NRS values composing the PsAID is missing, the imputation is as follows:
calculate the mean value of the 11 other (non-missing) NRS (range, 0-10)
impute this value for the missing NRS
Then, calculate the PsAID as explained above.

If 2 or more of the NRS are missing, the PsAID is considered as missing value (no imputation).
